# Cochlear Implantation in Patients with Neurofibromatosis Type 2 and Patients with Vestibular Schwannoma in the Only Hearing Ear

**DOI:** 10.1155/2012/157497

**Published:** 2012-02-28

**Authors:** Erika Celis-Aguilar, Luis Lassaletta, Javier Gavilán

**Affiliations:** ^1^Departamento de Otorrinolaringología, Universidad Autónoma de Sinaloa, 80030 Culiacán, SIN, Mexico; ^2^Department of Otolaryngology, “La Paz” University Hospital, 28046 Madrid, Spain

## Abstract

Cochlear implants are a new surgical option in the hearing rehabilitation of patients with neurofibromatosis type 2 (NF2) and patients with vestibular schwannoma (VS) in the only hearing ear. Auditory brainstem implant (ABI) has been the standard surgical treatment for these patients. We performed a literature review of patients with NF2 and patients with VS in the only hearing ear. Cochlear implantation (CI) provided some auditory benefit in all patients. Preservation of cochlear nerve integrity is crucial after VS resection. Results ranged from environmental sound awareness to excellent benefit with telephone use. Promontory stimulation is recommended although not crucial. MRI can be performed safely in cochlear implanted patients.

## 1. Introduction

### 1.1. Treatment Approach in Bilateral Hearing Loss

Bilateral hearing loss represents a great disability for patients with neurofibromatosis type 2 (NF2) and patients with vestibular schwannoma (VS) in the only hearing ear. The treatment for VS patients (unilateral and bilateral) is diverse, including observation, surgery, and radiotherapy [1].

**Figure 1:**
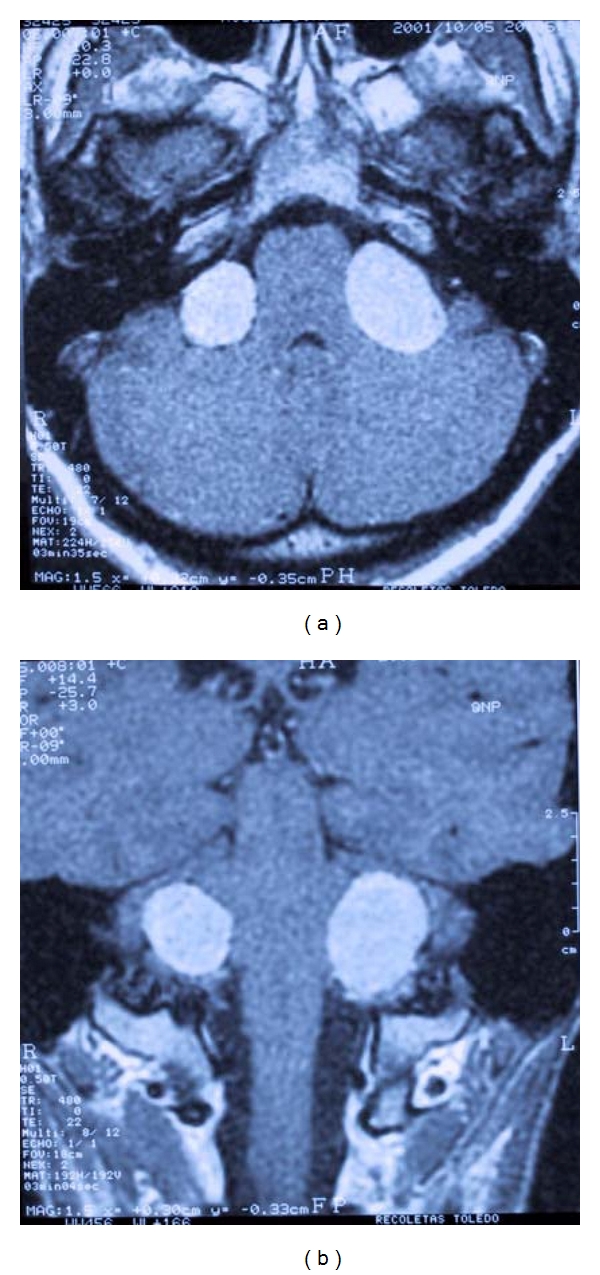
MRI axial and coronal view of neurofibromatosis type 2 patient.

Treatment of the worst hearing ear in NF2 patients could leave an only hearing ear with tumor and the risk of further hearing loss. Moreover, treatment of VS in an only hearing ear can also lead to deafness. Both situations represent a therapeutic dilemma [2]. Developments of new hearing rehabilitation strategies have changed the management of these patients.  Figure 1 shows the MRI of a patient with bilateral vestibular schwannomas.

### 1.2. Surgical Options in Hearing Rehabilitation

Since 1979, auditory brainstem implant (ABI) has been the only auditory rehabilitation option in patients with no serviceable hearing and previous schwannoma resection. ABI was designed to restore hearing in patients with nonfunctional cochlear nerves who were not candidates for cochlear implantation [3]. Although ABI provides environmental sound and significant lip reading assistance, they have not reached consistent results in speech discrimination [3–7].

Recently cochlear implants have emerged as a reasonable therapeutic option in selected cases. Initially patients should undergo schwannoma resection with preservation of the cochlear nerve as the main goal. Subsequently CI is done in a standard fashion through a cochleostomy or directly through the round window. Results of CI in NF2 patients and VS in an only hearing ear are quite promising [8–10] and may provide outcomes comparable to those of postlingually implanted nontumor patients.

In this study we reviewed cochlear implantation as hearing rehabilitation in patients with NF2 and in patients with VS in the only hearing ear.

### 1.3. Cochlear Nerve Preservation and Function

Bilateral vestibular schwannomas in a NF2 patient can invade and grow within the cochlear nerve, while unilateral sporadic vestibular schwannoma (VS) only compresses it [11]. Also, identification of the surgical plane between the tumor and the cochlear nerve is more demanding on an NF2 patient than on sporadic VS [11, 12]. Despite the difficulty implied in schwannoma resection, there are reports that demonstrate cochlear nerve preservation after surgery. Different approaches used for tumor removal are retrosigmoid, middle fossa and translabyrinthine [8–10, 12]. Radiosurgery is also an option for VS treatment previous to CI [13].

Cochlear nerve function is not always preserved in spite of anatomic preservation of the cochlear nerve. An intact nerve does not necessarily mean a normal nerve histologically [14]. Cochlear nerve can be injured during surgery, causing intraneural hemorrhage, disruption of axons, especially in the Obersteiner-Redlich zone. A moderate injury can cause hearing loss but still allows electrical transmission of the stimulus. One way to evaluate function of the nerve is through electrical promontory stimulation.

### 1.4. Spiral Ganglion Cells and Deafness

Spiral ganglion cells are the neural structure stimulated by the cochlear implant, therefore crucial for cochlear implantation [15].

With deafness there is a loss of hair cells with subsequent degeneration of spiral ganglion cells [16]. Some authors have suggested that this is not necessarily true. Teufert et al. [17] in a temporal bone study from the House Clinic stated that in contrast with animal studies spiral ganglion cells can survive even in the absence of hair cells. They demonstrated that 16 of 33 ears had peripheral processes despite the total absence of hair cells.

Nadol found [15] in 93 temporal bones of profoundly deaf people a diminished main spiral ganglion population. The loss of spiral ganglion cells was greater in older patients, with longer duration of hearing loss and located in the basal turn. An association with etiology was also found. Patients with aminoglycoside toxicity and sudden idiopathic deafness had the highest spiral ganglion cell counts, whereas patients with the lowest counts were diagnosed with postnatal viral labyrinthitis, congenital deafness, or bacterial meningitis. The authors described 27,304 ± 4,203 spiral ganglion count in the normal hearing group and 15,463 ± 7,838 in patients with hearing loss (*P* = 0.0008).

Fayad et al. published that 3500 intact neurons were required for a cochlear implant to be successful [18]. Useful auditory sensation could result from 10% of the total number of ganglion cells.

 There is controversy on the role spiral ganglion cells have on speech perception [15, 19]. Fayad et al. in 1991 compared the speech results in single channel implanted patients. No significant correlation was found between the perceptual scores and ganglion cell numbers [18]. Two patients with 3000 surviving neurons performed similarly more than patients with 15000 neurons. Blamey [19] also found no evidence of strong association between speech perception and ganglion cell number. The authors assumed that the minimum number of cells required for speech perception is quite low. A histopathological study [15] demonstrated a poor correlation between speech performance and number of ganglion cells.

### 1.5. Labyrinthectomy and Spiral Ganglion Cells Integrity

Histological studies in temporal bones with labyrinthectomy have demonstrated that enough ganglion cells may survive, proving the feasibility of cochlear implants [20, 21]. Facer et al. [22] reported a case who underwent labyrinthectomy and cochlear implantation with beneficial results. Another temporal bone study [23] examined 8 specimens from patients who underwent acoustic tumor removal through a translabyrinthine approach. Because of different histological cochlear modifications after surgery (e.g.: ossification, fibrosis), they recommended simultaneous cochlear implantation for better results.

### 1.6. Cochlear Implantation and Neural Integrity

The effect of electrical stimulation on residual ganglion cells is also controversial. Coco et al. [24] showed a significant increase in the soma area of ganglion cells adjacent to the stimulating electrodes in cats with profound hearing loss and chronic electrical stimulation. The increased in soma area was explained as a possible increase in cell biosynthesis. A significant rescue of ganglion cells in middle and apical turns of stimulated cochlea in animals with partial hearing was also found [24]. Linthicum et al. [25] demonstrated no modification in spiral ganglion cell population with prolonged electrical stimulation (up to 14 years).

In the evaluation of trauma to the inner ear by cochlear implantation, Nadol [15] found damage to the lateral cochlear wall and basilar membrane in the upper basal turn. New bone formation and perielectrode fibrosis was common. Degeneration of the spiral ganglion were not correlated with cochlear changes.

### 1.7. Status of the Cochlear Nerve after Hearing Preservation Techniques

Hearing preservation approaches include middle fossa and retrosigmoid. In spite of preservation of the cochlear nerve during surgery, several patients lose hearing in the postoperative period. Theories that explain this finding [26] are direct neuronal disruption of the cochlear nerve and vascular injury to the cochlea. As stated before, promontory electrical stimulation can be used to assess ganglion cell survival. An electrical response can be obtained if 10–25% of spiral ganglion cells are preserved. Cueva et al. [26] studied 6 patients with anacusis following acoustic neuroma surgery. Only one patient had subjective auditory perception with EPS. A possible explanation of anacusis in this case was vascular compromise of the cochlea.

 Neff et al. [8] also reported cases with postoperative hearing loss with hearing preservation techniques in spite of anatomically preserved cochlear nerve. They suggested cochlear implantation in these patients even if blood supply is interrupted as long as electrical promontory stimulation (EPS) is positive.

 Failure of hearing preservation after surgery has also been described by Lustig et al. [9] who included 5 cases with this situation. Patients with good performance with cochlear implantation were supposed to have vascular injury and those with poor performance a significant neuronal injury due to tumor growth or surgical removal. All patients in this series had some functional improvement with cochlear implantation, emphasizing that even limited electrical stimulation can offer benefit in these patients.

McKenna et al. [27] did a long-term followup (3.4 to 10.4 years) of patients with VS removal via RS approach. Four patients (22%) experienced a decline in PTA or SDS, probably related to vascular compromise or microscopic recurrence. Neff et al. [8] in contrast, refuted that hearing deteriorated after a certain postoperative period. This group reported the largest followup in patients with cochlear implantation and VS resection available in the literature. With a mean followup of 7.9 years, in all but one case, hearing results did not deteriorate over time.

## 2. Methods

 We performed a literature review from 1992 to 2010 by using a comprehensive search strategy in Cochrane library, MEDLINE, and PubMed databases.

 The following search terms were used:* neurofibromatosis type 2, vestibular schwannoma, acoustic neuroma, bilateral hearing loss, deafness, auditory rehabilitation, auditory brainstem implants, radiosurgery, and MRI. *The limiting search terms were* cochlear implantation, treatment, and clinical cases*. Additional articles were identified by hand searching the references from original and review articles. The search was restricted to English and Spanish language.

 The identified articles were assessed for eligibility and only the articles that explored cochlear implantation in NF2 patients and VS in the only hearing ear were selected. Only retrospective case series or case reports were available for review. All patients underwent cochlear implantation *after* VS resection and/or radiosurgery. Diverse outcome assessments were reported.

## 3. Reported Outcomes in Patients Undergoing Cochlear Implantation and VS Resection

Various centers have reported their outcomes in cochlear implantation and vestibular schwannoma resection [8–10, 12, 28–36]. The majority included almost exclusively NF2 patients (see Tables 1–3). Twenty-seven patients were treated with VS surgery and cochlear implantation and 6 cases with radiosurgery and cochlear implantation. Age ranged from 15 to 84 years. As expected, the oldest patient was treated with radiosurgery.

 Only two studies reported two cases with unilateral vestibular schwannoma in the only hearing ear. A slight better hearing performance could be observed [28] in a unilateral vestibular schwannoma patient compared to the performance of an NF2 patient of the same institution (vowel identification 100%, bisyllable word recognition 95% versus vowel identification 80%, bisyllable word recognition 90%, resp.). Another case with VS in the only hearing ear was published by Arriaga and Marks [10]. This patient presented with a postoperative PTA of 25 dB, the best reported in the literature. However, no speech tests were reported. Lip reading with the implant was 50% better than without the implant with markedly improved recognition of environmental sounds and communication at home.

Regardless of the time of cochlear implantation or approach used, the speech perception outcome through the studies is diverse. It ranges from no benefit to significant better speech discrimination. It is important to point out the different outcomes described in these studies. Some studies focus on open-set speech perception whereas others report on closed-set tests. Some tests are assisted by lip reading. We detail the results in subsequent tables, to be analyzed separately (Tables 1 and 2).

Lustig et al. [9] reported the most contrasting results. Three patients who underwent VS resection and cochlear implantation had 0% in all the speech tests. However, all patients had environmental sound awareness, sound localization, and improved performance. The variability of the performance was explained by the authors, by variations in the status of the cochlear nerve. Another explanation was meaning serviceable contralateral hearing. Patients were assumed to have difficulty in integrating input from a cochlear implant from the deaf ear with contralateral serviceable hearing. It is assumed that as patients lose hearing in the better ear they should rely more on the implanted side.

In the whole series, fourteen patients were reported to use the telephone. Most of them belong to the VS resection group (Table 1). Only one patient (1/6) was described as a telephone user in the radiosurgery group. This may be due to the lack of reported data in the radiosurgery patients.

The daily use of the cochlear implant was reported in 16 patients (48%).

There are 7 cases of simultaneous cochlear implantation and vestibular schwannoma resection. Theoretically, a prompt implantation will reduce cochlear fibrosis or ossification and avoid multiple interventions. Overall, the authors reported good results compared to their counterparts (delayed surgery). All patients who underwent simultaneous implantation had a translabyrinthine schwannoma resection. Vincenti et al. [29] found no cochlear ossification within 3 months after TL VS removal. This was the only case of postponed cochlear implantation in TL surgery. In another case [28] from another institution, cochlear implantation was attempted one year after TL surgery. The authors found total ossification and implantation could not be performed. Due to bilateral hearing loss secondary to NF2, they decided to resect the contralateral VS with simultaneous CI in that side, which was performed uneventfully.

Translabyrinthine approach did not affect speech reception results and outcomes compared with other approaches.

The longest period between surgery and implantation was 84 months following a retrosigmoid approach, with reported hearing gain.

The longest followup was done by Neff et al. [8], with an average of 7.9 years after CI.

### 3.1. Electrical Promontory Stimulation (EPS)

 To assess the status of the cochlear nerve, electrical promontory stimulation is recommended before implanting patients with VS. Promontory or round window stimulation evaluates the neurons in the cochlear spiral ganglion. Both anatomically and functionally preserved cochlear nerves are important requirements for implantation. However, false negative stimulations have been described if EPS is performed too close to the surgical event. It is recommended to repeat measurements 6–8 weeks postoperatively, even after a previous negative measurement [8].

In the literature review we found four cases with promontory stimulations initially negative and subsequently positive (Tables 1 and 2). The possible mechanism of this phenomenon is described by the authors [8, 12, 34]. One theory states possible surgical damage to the cochlear nerve with postoperative inflammation that subsequently subsides. Another theory highlights the role of vascular supply to the cochlear nerve. Vincenti et al. [29] reported a case in which the EPS was negative, and because cochlear nerve integrity was certain, they proceeded with the cochlear implant. They had environmental sound recognition and usefulness lip reading. Another case with negative EPS was published by Trotter and Briggs [13] after radiosurgery, the results showing improved outcome with 72% in CUNY sentences in quiet and 45% in CNC words.

Cueva et al. [26] reported 6 cases with anacusis following VS surgery. Only one patient had postoperative positive EPS suggesting vascular compromise to the cochlea (especially stria vascularis) with a partially intact cochlear nerve.

 The majority of the studies reviewed (Table 1) reported positive electrophysiological studies consisting of electrical promontory stimulation (EPS) or compound action potential (CNAP). Results were recorded simultaneously and up to 84 months postsurgically.

 Authors considered the performance of electrophysiological tests an important examination prior to implantation, although not crucial. Thirteen cases of the present review did not have data on electrophysiological tests and were nonetheless submitted to cochlear implantation. These patients had good auditory benefit. Arístegui and Denia [28] reported one case with 100% in daily words and 100% in CID. Other cases had no reported EPS or speech tests but authors reported subjective auditory benefit. In addition to two cases with negative EPS, a total of 15 cases underwent CI without objective evidence of cochlear nerve function. This did not alter their final results.

### 3.2. Comparative Results with Cochlear Implantation in Conventional Postlingually Deaf Adults

 The speech tests results in conventional postlingually deaf adults undergoing conventional CI are similar with all devices manufactured. Good results in speech discrimination achieved by these patients are published through the literature [37–42], in general: 80 to 90% in vowel recognition [40], 50–60% in bisyllable identification [40], 70 to 100% in word recognition [41], 65% to 80% in correct sentence recognition scores [37–39], 85% HINT in quiet score, and 65% in HINT in noise score [42]. Two factors implied in good performance were age at implantation and duration of deafness.

 The House Clinic reported that up to one-third of postlingual adults undergoing implantation achieve open-set speech recognition [3]. Poor performers benefit from environmental sounds awareness and assisted lip reading skills.

Vincenti et al. [29] highlight the similarity of speech tests results between CI patients after VS resection compared with conventional postlingually implanted patients. The larger case series (Table 1) reported good speech results. Neff et al. [8] described HINT results ranged from 83 to 96%, CID between 22–100%. On the other hand, Arístegui and Denia [28] found 100% daily words recognition and 100% in CID in two postimplanted patients with VS resection. Tran Ba Huy et al. [30] described the results of open sentences recognition test without lip reading from 81–97%, although Lustig et al. [9] reported more variable results when 3 of their patients performed 0% in all speech tests.

The best results in speech discrimination achieved by postimplanted patients with NF2 or patients with VS in the only hearing ear are comparable with the results obtained by conventional postlingually deaf adults.

### 3.3. Comparative Results with ABI

Colletti et al. (2009), compared open-set speech perception in tumor and nontumor patients treated with ABI [7]. They found 10–100% on open-set speech perception scores in nontumor patients, compared with 5–31% in tumor patients. This difference was statistically significant. The authors conclude that ABI is an effective tool for hearing rehabilitation in patients with profound hearing loss who cannot be fitted with cochlear implants (CI).

Grayeli et al. [4] reported open set dyssyllabic word recognition of 36% for vision only mode, 3% for sound only mode, and 65% for vision plus sound mode in NF2 patients with ABI.

In the House Clinic review on ABI, 85% of patients perceived auditory sensation [3]. Combined with lip reading cues, 93% of patients improved sentence understanding in 3 to 6 months. Most patients had environmental sound awareness and understanding of closed set words, consonants, and vowels, while open set speech discrimination was difficult to achieve. In contrast, Lenarz et al. [6] reported better results with 2 out of 11 patients achieving some kind of telephone conversation. In general open set speech recognition in the auditory mode alone is not common among patients implanted with ABI [5, 6].

The only study found to compare ABI versus CI after VS resection from the same institution was performed by Vincenti et al. [29]. They included 9 patients, 4 with CI and 5 with ABI. At 1-year followup, the performance in close set was similar. Nonetheless, the open set tests showed a compelling difference. Mean common phrases comprehension score was 60% in the CI group and 29% in the ABI group. Mean sentence recognition score was 55% in the CI group and 27% in the ABI patients. Moreover, mean bisyllabic word recognition score was 53% in the CI group and 32% in the ABI group. The authors concluded that when possible the CI should be the preferred hearing rehabilitation device for patients with VS, not only because of the better hearing results, but also because of the reduction of the surgical risks and lesser extent of the operation.

As poor speech reception results are prevalent in the ABI literature, it is recommended to preserve the cochlear nerve during VS resection in order to achieve better hearing results with a cochlear implant.

## 4. Reported Outcomes in Patients with VS Treated with Radiosurgery and Cochlear Implantation

 Radiosurgery is an alternative to surgery for patients with VS. This treatment consists in the delivery of ionizing radiation to the intracranial target with the use of a stereotactic technique [43, 44].

Irradiation of head and neck tumors without ear involvement has shown to affect ganglion cell population in a temporal bone study [45]. Marked spiral ganglion loss in the basal turn of the cochlea was found in the irradiated group compared with the same area in controls. Bohne et al. [46] studied in a chinchilla model the effect of fractionated radiation on the ear. They found degeneration of hair cells, supporting cells, and cochlear neurons. Guinea pigs also showed damaged to outer and inner hair cells after fast neutron irradiation greater than 15 Gy [47].

 In relation to gamma knife surgery, Linskey et al. described the radiation exposure of normal temporal bone structures during gamma knife surgery [48]. A mean of 5.5 Gy absorption was found at the inferior portion of the basal turn of the cochlea and 8.9 Gy absorption at the modiolus of the basal turn. Although doses greater than 12 Gy are described as potentially toxic to the inner ear, the basal turn of the cochlea absorbed doses greater than 12 Gy in 10 to 14% of cases. Additionally a change in PTA was significantly poorer at 12 months for patients whose cochlea received 4.75 Gy or more [49].

Wackym et al. [50] examined 59 patients and found a hearing loss pattern consistent with stria vascularis devascularization after gamma knife surgery, that is, hearing loss across all frequencies and relative preservation of speech discrimination ability. No neural hearing loss was registered. Based on this data, direct cochlear damage could be the sole cause of post radiotherapy hearing loss [50]. Furthermore, they did not find a correlation between change in tumor size after radiotherapy and hearing loss, making tumor edema a less likely cause. In order to confirm this, more studies are required, including temporal bone histopathology examination.

Among series describing cases with cochlear implantation after radiation are those of Lustig, Tran Ba Huy et al., and Trotter and Briggs. Lustig et al. [9] reported 2 cases treated with radiosurgery and then cochlear implantation. These 2 cases had the best speech test results at that same institution (one patient MTSr 46%, the other SDS 46%, HINT 98%). The authors did not explain this finding.

Tran Ba Huy et al. and Trotter and Briggs [13, 30] reported good results in speech score tests after cochlear implantation and radiosurgery in 3 more patients. Mean followup was 32 months with no sign of malignancy until the end of followup (Table 3).

## 5. MRI Followup in Cochlear Implanted Patients

 Between the cochlear implant and the MRI there is a magnetic field interaction. CIs have internal magnets which interact with magnets contained in the MRI scanner [51].

 An important issue in NF2 patients and patients with resected VS is the necessity of followup with MRI.

 To perform MRI, there are basically 3 options: removal of the magnet before MRI, implantation of magnetless device, and implementation of low Tesla's MRI.

 The risks of MRI imaging in patients with the magnet device in place include potential device displacement, overheating of the surrounding tissues, demagnetization of the device, and cochlear implant malfunction.

 CI magnets described to be removable by a surgical procedure are Cochlear Corporation CI24M, CI24R, Nucleus Freedom, CI24ABI devices, and Advanced Bionics HiRes90K [51]. This option requires two minor surgical procedures, one to remove the magnet and another to replace it.

 Current US FDA guidelines have approved the use of 0.2 to 1.5 Teslas with the magnet in place in pulsar and sonata MED-EL Corp [51]. Unfortunately, 0.2 or lower than 1.0 Tesla MRI is not available in most hospitals, while 1.5 Tesla is becoming more universally used. Ex vivo and in vivo studies recommend the use of 1.5 Teslas for a safe MRI scan in patients with cochlear implant [52, 53].

 Baumgartner et al. [54] published the results of 30 cochlear implants undergoing 1.0 Tesla MRI examination. No adverse effects were reported. They recommended scanning Med El Combi 40 and Nucleus mini 22 series at 1 Tesla as a safe procedure, magnet removal being not necessary.

 During a 1.5 Tesla MRI scan, the device is subject to forces that can potentially cause its movement. The force described with the magnet removed is 0.03 N in a 1.5 Tesla MRI. On the other hand, with the magnet in place the force is 0.42 N. However, the force described to fracture the CI receiver bed was much higher than that generated by a 1.5 Tesla MRI [52].

Deneuve et al. [55] reported a case of magnet displacement during 1.5 T MRI. Interestingly, the authors took all precautions and had an external fixation dressing at the time of the MRI. Other authors suggest that previous surgery to remove and replace the magnet in this patient may have weakened the pocket [52].

 Heating of the CI during 1.5 Tesla MRI is reported to be lower than 0.1°C [52] and the electrical stimulation is less than the intensity required for auditory stimulation. In an ex vivo study, a temperature lower than 1.0°C was measured in a 3.0 Tesla MRI. A maximum temperature of 2.0°C is set by the industry standard as the temperature that can damage the tissues and cause patient discomfort [51].

 Cadaver studies have shown no important demagnetization of the CI during 1.5 Tesla MRI [51]. The use of compression dressings is recommended for external fixation of the CI [52].

 The magnetic field interactions depend on geometric orientation of the poles. Majdani et al. [51] reported that demagnetization depends on the angle between the magnetic field of the CI magnet and the MRI. Important demagnetization happened if this angle was greater than 80 degrees. Thus, excessive head turning is to be avoided during MRI scan.

 Another issue to consider is the image quality obtained from the MRI scan. While in the contralateral side of the cranium and remainder of the body, the image has no distortion; ipsilateral soft tissues within 7 to 8 cm from the magnet are poorly visualized [52]. Some authors recommend combining MRI with high resolution CT with contrast to counterweigh the area of image distortion.

 An ex vivo study performed MRI tests (1.5 Tesla and 0.3 Tesla) to a Clarion 1.2 magnet-containing cochlear implant. The area of distortion had a radius of 60 mm in the 1.5 Tesla MRI group and 100 mm in the 0.3 Tesla group. There was no detectable temperature increase in both groups [56].

**Figure 2:**
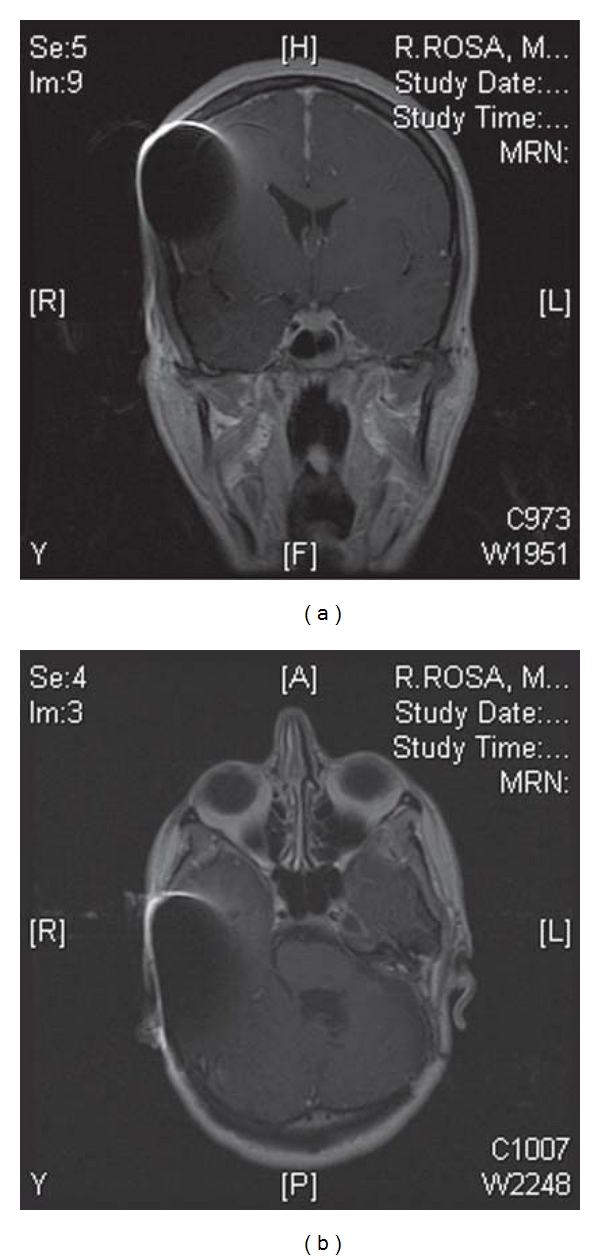
MRI followup at 1.5 T of a patient with surgically removed VS and cochlear implantation (Pulsar CI 100, Med-el).

 Furthermore, a retrospective study by the John Hopkins University included 16 patients with CI from 3 major manufactures. All patients underwent 1.5 Tesla MRI. They reported an artifact on brain MRI with a maximal anterior-posterior dimension of 6.6 cm and a lateral dimension of 4.8 cm around the magnet device with no difference among the 3 CI manufacturers. The contralateral internal auditory canal was seen in all patients and the ipsilateral internal auditory canal was at least partly visible in all but one patient. No magnet displacement was observed. They recommended the use of 1.5 Teslas MRI in CI patients [52]. On the other hand, Baumgartner et al. in a 1.0 Tesla MRI setting reported minimal artifact although they provided no artifact area measurements and it was not clear whether the ipsilateral IAC was evident on MRI [54]. See Figure 2 for an example of MRI followup in an implanted patient.

 A safe use of 1.5 T MRI is recommended for cochlear implant patients. In patients with NF2 and VS, ipsilateral MRI control can be accomplished with adjuvant CT with contrast, and an artifact around 5 cm should be expected. Contralateral surveillance is achieved with no image distortions.

## 6. Conclusions

Cochlear implantation in patients with neurofibromatosis type 2 and patients with vestibular schwannoma in the only hearing ear is a reasonable hearing rehabilitation option. Early surgical intervention with preservation of the cochlear nerve should be considered. Auditory benefit can be expected in most patients, and in good candidates excellent benefit with good speech discrimination and telephone use may be achieved. Surgical resection through translabyrinthine approach does not affect speech test outcomes. Radiosurgery has good auditory results. Although not crucial, promontory stimulation for evaluating the integrity of the cochlear nerve is recommended. MRI up to 1.5 Teslas with external head fixation can be done safely in cochlear implanted patients.

## Figures and Tables

**Table 1:** Series report on cochlear implantation and vestibular schwannoma resection regardless on the approach used or timing between surgeries.

Author	Patients no.	Age	Approach	Radiation	Time to implantation (m)	Cause HL	Followup (m)	Cochlear implant PTA dB	Speech tests	Telephone	Daily use	EPT after VS resection (m)
Arístegui and Denia [28] 2005	1	52	TL	No	S	NF2	18	NA	VI 80%, BWR 90%, daily words 100%, CID 100%.	Yes	NA	NA
	2	45	TL	No	S	VS	6	NA	VI 100%, BWR 95%, daily words 100%, CID 100%	NA	NA	+

	1	35	NA	No	NA	NF2	28	55	0% all speech tests	NA	Yes	NA
	2	51	NA	No	NA	NF2	40	35	MTSr 6%, MTSc 79%	NA	Yes	NA
Lustig et al. [9] 2006	3	16	NA	No	NA	NF2	30	40	0% all speech tests	NA	Yes	NA
	4	28	NA	No	NA	NF2	88	45	0% all speech tests	NA	Yes	NA
	5	57	NA	No	NA	NF2	9	30	Phonemes 35%, HINT 21%	NA	Yes	NA

	1	15	MF	No	9	NF2	156	NA	3SWLOS 45%, 3SWLCS 75%	Yes	NA	EPS 6 m +
	2	26	RS	No	2	NF2	108	NA	HINT 91%, CID 98%, CUNY45%	Yes	NA	**EPS 2 m + **
Neff et al. [8] 2007	3	53	RS	No	72	NF2	60	NA	HINT 96%, CID 100%, CUNY 93%, 4CS 100%	Yes	NA	EPS 12 m +
	4	37	RS	No	Few m	NF2	98.4	NA	HINT 83%, CID 90%, 4CS 100%	Yes	NA	**EPS 2 m + **
	5	37	TL	No	S	NF2	61.2	NA	HINT 96%, CID 100%, 4CS 100%	Yes	NA	NA
	6	31	RS	No	1.5	NF2	84	NA	CID 22%, 4CS 85%, CUNY 0%	No	NA	EPS 1.5 m +

	1	47	RS	No	3	NF2	12	NA	VI 100%, CI 100%, BWR 80%, SR 90%, CPC	Yes	Yes	EPS +
Vincenti et al. [29] 2008	2	24	TL	No	S	NF2	12	NA	100% VI 100%, CI 100%, BWR 72%, SR 81%, CPC 86%	Yes	Yes	CNAP, S +
	3	32	TL	No	3	NF2	12	NA	VI 100%, CI 100%, BWR 50%, SR 50%, CPC 55%	Yes	Yes	EPS +
	4	36	RS	No	3	NF2	12	NA	VI 40%, CI 39%, BWR 10%, SR 0%, CPC 0%	No	Yes	CNAP−

Tran Ba Huy et al. [30] 2009	1	41	RS	No	4	NF2	24	NA	OSWSLR 90%, OSWSWLR 68%, OSSLR 89%, OSSWLR 81%, OSSWLRWN 6%, PCC 89%, PWCC 30%	Yes	NA	EPS 2 m +
	2	17	TL	No	S	NF2	12	NA	OSWSLR 97%, OSWSWLR 87%, OSSLR 99%, OSSWLR 97.8%, PCC 89%, PWCC 60%	Yes	NA	NA

TL: translabyrinthine; NA: nonavailable; MF:middle fossa; RS: retrosigmoid; S: simultaneous; NF2: neurofibromatosis type 2; VS: vestibular schwannoma; 3SWLOS: three syllabic word list open set; 3SWLCS: 3 syllabic word list closed set; HINT: hearing in noise sentence testing; CID: Central Institute for the Deaf sentences of everyday speech; CUNY: City University of New York sentences in noise; 4CS: four-choice spondee (closed set); MTSr: Monosyllable Trochee Spondee recognition; MTSc: Monosyllable Trochee Spondee category; SDS: speech discrimination score; VI: vowel identification; CI: consonant identification; BWR: bisyllable word recognition; SR: sentence recognition; CPC: common phrases comprehension; OSWSLR: open-set word score with lip reading; OSWSWLR: open set word score without lip reading; OSSLR: open set sentences with lip reading; OSSWLR: open set sentences without lip reading; OSSWLRWN: open-set sentences without lip reading with noise; PCC: phone with contextual cues; PWCC: phone without contextual cues; EPT: electrical promontory test; EPS: electrical promontory stimulation; CNAP: compound action potential. Bold letters: patients who initially had negative promontory stimulation.

**Table 2:** Case reports on cochlear implantation and vestibular schwannoma resection regardless of the approach or timing between surgeries.

Author	Patients no.	Age	Approach	Radiation	Time to implantation (m)	Cause HL	Followup (m)	Cochlear implant PTA dB	Speech tests	Telephone	Daily use	EPT after VS resection (m)
Hoffman et al. [12] 1992	1	30	RS	No	4	NF2	12	NA	SRT 53 dB SPL, SDT 43 dB SPL. MAC open set scores: spondees 40%, NU-6 word score 32%, NU-6 phoneme 53%, CID 72%.	Yes	NA	**EPS 2 m +**

Arriaga and Marks [10] 1995	1	65	TL	No	S	OT,VS	10	25	NA	NA	Yes	NA

Hulka et al. [31] 1995	1	31	RS	No	2	NF2	3	35	NA	NA	Yes	EPS 1.5 m +

Tono et al. [32] 1996	1	48	MF	No	15	NF2	17	NA	Tests with lipreading: MR 60%, WR 70%, SR 81%. Tests without lip reading: MR 24%, WR 62%, SR 43%.	Yes	NA	EPS 1 m +

Temple et al. [33] 1999	1	15	MF	No	9	NF2	12	NA	SRos 100%, 2WPR 70%, 3-4WPR 60%.	NA	NA	EPS 1.5 m +

Graham et al. [34] 1999	1	34	RS	No	84	NF2	10	NA	CUNY with lip reading 97%, BKB 34%, ESR 63%.	NA	Yes	**EPS 84 m +**

Ahsan et al. [35] 2003	1	53	TL	No	S	NF2	NA	NA	NA	NA	NA	NA

Nölle et al. [36] 2003	1	16	RS	No	24	NF2	24	NA	SRos: 88%	NA	NA	EPS +

TL: translabyrinthine; NA: nonavailable; MF: middle fossa; RS: retrosigmoid; S: simultaneous; NF2: neurofibromatosis type 2; VS: vestibular schwannoma; SRT: speech reception threshold; SDT: speech detection threshold; MAC: minimal auditory capabilities; NU: Northwestern University; CID: Central Institute for the Deaf sentences of everyday speech; MR: monosyllables recognition; WR: word recognition; SR: sentence recognition; EPT: electrical promontory test; EPS: Electrical promontory stimulation; CNAP: compound action potential; SRos: sentence recognition open set; 2WPR: 2 word phrases recognition; 3-4WPR: three-to-four word phrases recognition; CUNY: City University of New York sentences in noise; BKB: BKB sentences in quiet without lip reading; ESR: environmental sound recognition. Bold letters patients who initially had negative promontory stimulation.

**Table 3:** Case reports of patients with radio surgery for VS and cochlear implantation.

Author	Patients no.	Age	Radiation	Rx dose	Time to implantation (m)	Cause HL	Followup (m)	Cochlear implant PTA dB	Speech tests	Telephone	Daily use	EPT after VS Radiosurgery (m)
Lustig et al. [9] 2006	1	41	Yes	NA	NA	NF2	17	55	MTSr 46%	NA	Yes	NA
	2	50	Yes	NA	NA	NF2	18	35	SDS 46%, HINT 98%	NA	Yes	NA

Tran Ba Huy et al. [30] 2009	1	20	Gamma	NA	24	NF2	48	NA	Phonemes 95%, CSWS 100%, OSWS 100%, OSSWLR 97%, OSSWLRWN 89%	Yes	NA	EPS 24 m +

	1	52	ST	54 Gy 30 fr	60	NF2	36	NA	CUNY sentences in quiet 96%, CUNY sentences in noise 79%, CNC words 79%	NA	Yes	EPS 60 m +
Trotter and Briggs [13] 2010	2	41	ST	50.4 Gy 28 fr	7	NF2	12	NA	CUNY sentences in quiet 72%, CNC words 45%	NA	NA	EPS 7 m−
	3	84	ST	12 Gy single fr	12	NF2	NA	NA	NA	NA	Yes	NA

NA: nonavailable; NF2: neurofibromatosis type 2; VS: vestibular schwannoma; ST: stereotactic radiation; Gy: gray unit; fr: fraction; MTSr: Monosyllable Trochee Spondee recognition; SDS: speech discrimination score; HINT: hearing in noise sentence testing; CSWS: closed set word score; OSWS: open-set word score; OSSLR: open set sentences with lip reading; OSSWLR: open set sentences without lip reading; OSSWLRWN: open-set sentences without lip reading with noise; EPT: electrical promontory test; EPS: electrical promontory stimulation; CUNY: City University of New York sentences; CNC: consonant nucleus consonant word lists.
